# PP13, Maternal ABO Blood Groups and the Risk Assessment of Pregnancy Complications

**DOI:** 10.1371/journal.pone.0021564

**Published:** 2011-07-25

**Authors:** Nandor Gabor Than, Roberto Romero, Hamutal Meiri, Offer Erez, Yi Xu, Federica Tarquini, Laszlo Barna, Andras Szilagyi, Ron Ackerman, Marei Sammar, Tibor Fule, Katalin Karaszi, Ilona Kovalszky, Zhong Dong, Chong Jai Kim, Peter Zavodszky, Zoltan Papp, Ron Gonen

**Affiliations:** 1 First Department of Obstetrics and Gynecology, Semmelweis University, Budapest, Hungary; 2 Wayne State University, Detroit, Michigan, United States of America; 3 Diagnostic Technologies Ltd., Yokneam, Israel; 4 TeleMarpeh Ltd., Tel Aviv, Israel; 5 Department of Obstetrics and Gynecology “B”, Soroka University Medical Center, Ben Gurion University of the Negev, Beer Sheva, Israel; 6 Institute of Enzymology, Hungarian Academy of Sciences, Budapest, Hungary; 7 Department of Biotechnology Engineering, ORT Braude College, Karmiel, Israel; 8 First Department of Pathology and Experimental Cancer Research, Semmelweis University, Budapest, Hungary; 9 Department of Obstetrics and Gynecology, Faculty of Medicine, Bnai Zion Medical Center, Technion - Israel Institute of Technology, Haifa, Israel; Institute of Zoology, Chinese Academy of Sciences, China

## Abstract

**Background:**

Placental Protein 13 (PP13), an early biomarker of preeclampsia, is a placenta-specific galectin that binds beta-galactosides, building-blocks of ABO blood-group antigens, possibly affecting its bioavailability in blood.

**Methods and Findings:**

We studied PP13-binding to erythrocytes, maternal blood-group effect on serum PP13 and its performance as a predictor of preeclampsia and intrauterine growth restriction (IUGR). Datasets of maternal serum PP13 in Caucasian (n = 1078) and Hispanic (n = 242) women were analyzed according to blood groups. *In vivo*, *in vitro* and *in silico* PP13-binding to ABO blood-group antigens and erythrocytes were studied by PP13-immunostainings of placental tissue-microarrays, flow-cytometry of erythrocyte-bound PP13, and model-building of PP13 - blood-group H antigen complex, respectively. Women with blood group AB had the lowest serum PP13 in the first trimester, while those with blood group B had the highest PP13 throughout pregnancy. In accordance, PP13-binding was the strongest to blood-group AB erythrocytes and weakest to blood-group B erythrocytes. PP13-staining of maternal and fetal erythrocytes was revealed, and a plausible molecular model of PP13 complexed with blood-group H antigen was built. Adjustment of PP13 MoMs to maternal ABO blood group improved the prediction accuracy of first trimester maternal serum PP13 MoMs for preeclampsia and IUGR.

**Conclusions:**

ABO blood group can alter PP13-bioavailability in blood, and it may also be a key determinant for other lectins' bioavailability in the circulation. The adjustment of PP13 MoMs to ABO blood group improves the predictive accuracy of this test.

## Introduction

ABO blood-group antigens are oligosaccharides attached to cell-surface glycoconjugates expressed by epithelia, endothelia and erythrocytes (RBCs) in primates [1], [2]. Although their function has not yet been revealed, ABO antigens might have been evolutionarily advantageous in conferring resistance against pathogens [3]. The susceptibility to various diseases, such as infections, cancer, cardiovascular diseases and hematologic disorders, have been associated with ABO blood groups [3]–[10]. Interestingly, ABO blood group is a key determinant of coagulation factor VIII and von Willebrand factor plasma concentrations [4], [5]. Low plasma concentrations of these glycoproteins in blood-group O individuals may lead to excess bleeding, while elevated plasma concentrations of these factors in non-O blood-group individuals have been implicated in increasing the risk of thromboembolic and ischemic heart diseases [5]–[9]. Preeclampsia, a syndrome unique to human pregnancy and one of the leading causes of maternal and fetal morbidity and mortality [11], [12], is also associated with maternal blood group [13]–[15]. Patients with blood group AB have an increased risk of severe-, early-onset-, or intrauterine growth restriction (IUGR) associated forms of preeclampsia [14], [15].

Placental Protein 13 (PP13) is considered to be an early marker for preeclampsia [16]–[26]. It is a galectin (galectin-13) that binds beta-galactosides, such as N-acetyl-galactosamine, galactose, fucose, located at terminal positions on ABO blood-group antigens [27]–[29]. PP13 is primarily produced by the placenta in anthropoid primates [28]–[32] and is predominantly localized to the syncytiotrophoblast apical membrane, from where it can be secreted and/or shed into the maternal circulation [28]–[30], [32]–[34]. Our previous studies revealed its increased shedding from placental surfaces into maternal blood in patients with preterm severe preeclampsia and HELLP (hemolysis, elevated liver enzymes, low platelets) syndrome [33], [34], a phenomenon that may be responsible for elevated maternal serum PP13 concentrations in these patients in the second half of pregnancy [21], [33]. Of importance, decreased placental PP13 mRNA expression in these patients can be one of the underlying mechanisms leading to reduced first trimester maternal serum PP13 concentrations [33], [35], [36].

Although AB blood group and low first trimester maternal serum PP13 concentrations may separately be associated with increased risk of preeclampsia, we hypothesized that ABO blood group may affect PP13 bioavailability in maternal blood in normal and disease conditions. Indeed, PP13 may bind to beta-galactosides on ABO antigens and be sequestered on cell surfaces covered by these antigens similar to other galectins [37]–[39], and this phenomenon may affect maternal serum PP13 concentrations and the prediction accuracy of the PP13 test for pregnancy complications. Therefore, the objectives of this study were to 1) determine the relation between maternal serum PP13 and maternal blood groups throughout pregnancy; 2) confirm the differential binding of PP13 to RBCs of various ABO blood types; and 3) investigate whether the adjustment of maternal serum PP13 multiples of the medians (MoMs) to maternal blood groups could improve the predictive value of the PP13 test for preeclampsia and IUGR.

## Materials and Methods

### Ethics statement

The reported studies were approved by the Institutional Review Boards of the *Eunice Kennedy Shriver* National Institute of Child Health and Human Development (NICHD), National Institutes of Health (NIH), Department of Health and Human Services (DHHS, Bethesda, MD, USA) and the Sótero del Río Hospital (Santiago de Chile, Chile), the Maccabi Institutional Review Board (Israel), the Health Science Board of Hungary (Budapest, Hungary) and the Human Investigation Committee of Wayne State University (Detroit, MI, USA), respectively. Written informed consent was obtained from women prior to sample collection. Specimens were coded and data were stored anonymously.

### Determination of the effect of maternal blood groups on maternal serum PP13

#### Longitudinal and cross-sectional study on Caucasian patients

Gonen et al. [21] performed a prospective, longitudinal, multi-center study in Maccabi Healthcare Services, enrolling pregnant women with singleton pregnancy at prenatal community clinics in Israel. From the recruited 1366 women, 254 were excluded due to missed abortion (n = 95), non-compliance with the protocol (n = 32), or lack of blood-group information (n = 127). From the 1078 women included in this analysis, 20 patients developed preeclampsia (five complicated by IUGR), 52 patients had a fetus with IUGR, while 1006 women had pregnancies unaffected by these conditions. Patient characteristics are provided in Table S1. Maternal blood was obtained at 6–10, 16–20 and 24–28 weeks of gestation; sera were stored at −20°C and tested for PP13 with ELISA (Diagnostic Technologies Ltd, Yokneam, Israel). Intra- and inter-assay variations were 6.5% and 9.4%, respectively [21].

#### Cross-sectional study on Hispanic patients

Romero et al. [19] performed a nested case-control study on samples from a prospective, longitudinal study at the Perinatology Research Branch of the *Eunice Kennedy Shriver* National Institute of Child Health and Human Development (NIH, DHHS, USA), enrolling pregnant women with singleton pregnancy at the Sótero del Río Hospital (Santiago, Chile). Two hundred and forty-two normal pregnant women with blood-group information were included in this analysis. Patient characteristics are provided in Table S2. First trimester serum samples were collected between 8 and 13^+6^ weeks of gestation, stored at −80°C and tested for PP13 with ELISA (Diagnostic Technologies Ltd). Intra- and inter-assay variations were 7.3% and 19.5%, respectively [19].

#### Clinical definitions

Gestational age was determined by the last menstrual period and verified by crown rump length (CRL) [21] or by CRL and fetal biometry [19]. Preeclampsia was either defined [21] by the International Society for the Study of Hypertension in Pregnancy [40], or defined [19] by the Report of the National High Blood Pressure Education Program Working Group on High Blood Pressure in Pregnancy [41] and Sibai et al. [11]. IUGR was defined as birth-weight below the gestational age-specific 5th percentile according to local growth charts and birth-weight percentiles [42], [43].

### Determination of *in vivo* PP13-binding to RBCs

#### Placental tissue collection

PP13 immunostaining of RBCs was investigated in maternal and fetal blood spaces of placentas (n = 9) from normal pregnant women with no medical complications, delivering a term newborn with birth-weight appropriate for gestational age [44]. Placentas were collected at the First Department of Obstetrics and Gynecology (Semmelweis University, Budapest, Hungary, Federalwide Assurance: FWA00002527). Patients with a multiple pregnancy or a fetus having congenital or chromosomal abnormalities were excluded.

#### Construction of tissue microarrays (TMAs), PP13 immunohistochemistry and evaluation of immunostainings

Placentas were formalin-fixed, tissue blocks were paraffin-embedded and TMAs were constructed at the First Department of Pathology and Experimental Cancer Research (Semmelweis University) as described earlier [45]. After deparaffination and rehydration, endogenous peroxidases were inhibited with 10% H_2_O_2_. Slides were then incubated with 10 mM Tris-1 mM EDTA (pH 9.1; 30 min, 100°C) for antigen retrieval. Unspecific antibody binding was blocked (30 min; room temperature, RT) with NovoLink™ Polymer Detection System buffer (Novocastra Laboratories, Newcastle, UK). Slides were incubated (overnight; 4°C) with mouse monoclonal anti-PP13 antibody (clone 27-2-3; 1∶1000) in 1% bovine serum albumin (BSA) in PBS. After washing, the NovoLink kit was used for post-primary antibody blocking (30 min; RT). After repeated washing, incubation (30 min; RT) was performed with NovoLink (rabbit/mouse) polymer. Slides were then washed and developed with DAB Substrate Kit (Vector Laboratories, Burlingame, CA, USA), followed by hematoxylin counterstaining. In case of negative controls, the primary antibody was omitted. Slides were digitized with MIRAX DESK instrument (Zeiss, Gottingen, Germany) and analyzed with MIRAX TMA Module software (Zeiss). Images were deposited to a virtual laboratory (www.pathonet.org) and used for virtual microscopic evaluation (Mirax Viewer 1.11.49.0, Zeiss and 3DHistech Ltd., Budapest, Hungary).

### Determination of *in vitro* PP13-binding to RBCs

#### Recombinant protein production

Expression plasmids for PP13 and truncated PP13 (trPP13), which lacks the carbohydrate-binding domain (CRD), were constructed at the Perinatology Research Branch (NICHD, NIH, DHHS, Detroit, MI, USA) as described earlier [29]. *E. coli* M15 (Qiagen, Valencia, CA, USA) clone was transformed with these plasmids, grown in LB broth (100 ug/ml ampicillin; 50 ug/ml kanamycin; 37°C) until 0.8 OD_600 nm_ and then incubated with 1 mM IPTG (4 h). Cultures were centrifuged (6,000 *g*; 20 min), pellets were dissolved in 10 ml lysis buffer (Qiagen), and lysates were sonicated on ice and centrifuged (10,000 *g*; 20 min). Supernatants were incubated with 0.5 ml Ni-NTA beads (Qiagen; 1 h; RT), loaded onto 0.5 ml columns, and washed 2× with wash buffer (Qiagen). Recombinant PP13 and trPP13 were eluted with 2 ml elution buffer (Qiagen). The purity of elutes was verified by Coomassie blue staining after 4–15% gradient SDS-PAGE (Bio-Rad, Hercules, CA, USA).

#### PP13 binding assay and flow-cytometry

One milligram of PP13, trPP13 and BSA (Sigma-Aldrich, St Louis, MO, USA) were biotinylated with EZ-linkTM Sulfo-NHS-LC-Biotin (Pierce, Rockford, IL, USA), and then unconjugated biotin was dialyzed from the samples. Two million fresh, washed RBCs of different ABO blood types (Harper University Hospital Blood Bank, Wayne State University, Detroit, MI, USA) were incubated (4 h; 4°C) with biotinylated PP13 (0.175, 0.35, 0.7, and 1.4 uM aka 6.25, 12.5, 25 and 50 ug/ml in PBS), trPP13 (1.4 uM in PBS), and BSA (1.4 uM in PBS), or in PBS alone. RBCs were washed in PBS, incubated (2 h; 4°C) with AlexaFluor488-streptavidin (1∶200; Invitrogen-Molecular Probes, Carlsbad, CA, USA), and washed again in PBS. Cells were fixed in 1% paraformaldehyde (in PBS) and analyzed on BD FACSAria™ II with FACSDiva software (Franklin Lakes, NJ, USA). Fluorescence intensities were measured for 50,000 events per treatment in five independent experiments that were run in triplicate. PP13 binding affinity was derived from the mean fluorescence intensity.

### *In silico* modeling of PP13 - blood-group antigen binding

Amino acid sequences of 14 human galectins were aligned with MEGA 5 (http://megasoftware.net) to reveal sequence similarity in their CRDs. 3D model of PP13 complexed with blood-group H trisaccharid was built by superposing the structure of fungal galectin CGL2 complexed with blood-group H antigen (1ULD [46]) and the homology model of PP13 (1F87 [27]). Structural alignment was performed using TM-align (http://bioinformatics.buffalo.edu/TM-align), surface representation of PP13/blood-group H antigen complex was performed using GRASP2 (http://wiki.c2b2.columbia.edu/honiglab_public/index.php/Software:GRASP2).

### Statistical analyses

Maternal serum PP13 concentrations were not normally distributed; therefore, the Wilcoxon rank-sum test was used for group-comparisons. A stepwise multiple regression analysis was performed to reveal the correlation of covariates to PP13, including gestational age (GA), body mass index (BMI), ethnicity, smoking, maternal age, and parity. Possible significant interactions were evaluated by specifying a regression equation that included each individual covariate and any interaction between covariate-pairs. The following correlations were found in the Caucasian cohort [21]: GA, P<0.001; BMI, P = 0.099; ethnicity, P = 0.135; smoking, P = 0.497; maternal age, P = 0.07; parity, P = 0.204; BMI*ethnicity, P = 0.001; GA*BMI, P = 0.025. Correlations found in the Hispanic cohort [19] are the following: GA, P<0.001; BMI, P = 0.092; ethnicity, none (all Hispanic); smoking, P = 0.249; maternal age, P = 0.888; parity, P = 0.312; GA*BMI, P = 0.035.

PP13 concentrations were converted into gestational week-specific multiples of the medians (MoMs) among unaffected women [19], [21]. Gestational age-adjusted MoMs were sequentially adjusted to BMI, ethnicity, smoking, maternal age, and parity, and then further adjusted to ABO blood groups. Changes in PP13 concentrations and MoMs between the test periods were calculated as (X2-X1)/(W2-W1), where X1 and X2 were PP13 values at gestational weeks W1 and W2 [21]. Cross-sectional comparisons were performed with Kruskal-Wallis, Mann-Whitney, and Wilcoxon rank-sum tests.

The dataset used to ‘fit’ the regression models included individual subjects whose risk of preeclampsia we aimed to predict. To avoid potential bias due to ‘over-fitting’ of the models, the risk of preeclampsia for each woman was calculated using the ‘out of sample’ model in which values were calculated by running the analysis repeatedly, each time excluding one subject from the group. Sensitivities and specificities were calculated from PP13 MoMs for the disease groups (IUGR, preeclampsia and preeclampsia with IUGR) before and after adjustment for ABO blood groups. Receiver-operating characteristic (ROC) curves were generated to assess the test accuracy. The overall accuracy of the test was estimated with the area under the curves (AUCs). Data were analyzed using SAS® 9.1.3 (SAS Institute, Cary, NC, USA). A p<0.05 was considered statistically significant.

## Results

### Maternal serum PP13 bioavailability in pregnant women is dependent on ABO blood groups

To test whether maternal serum PP13 concentrations may be influenced by ABO blood groups, we re-analyzed published datasets on maternal serum PP13 in Caucasian [21] and Hispanic [19] populations.

#### Changes in maternal serum PP13 concentrations and MoMs according to maternal ABO blood group in Caucasian women [21]

**Figure 1:**
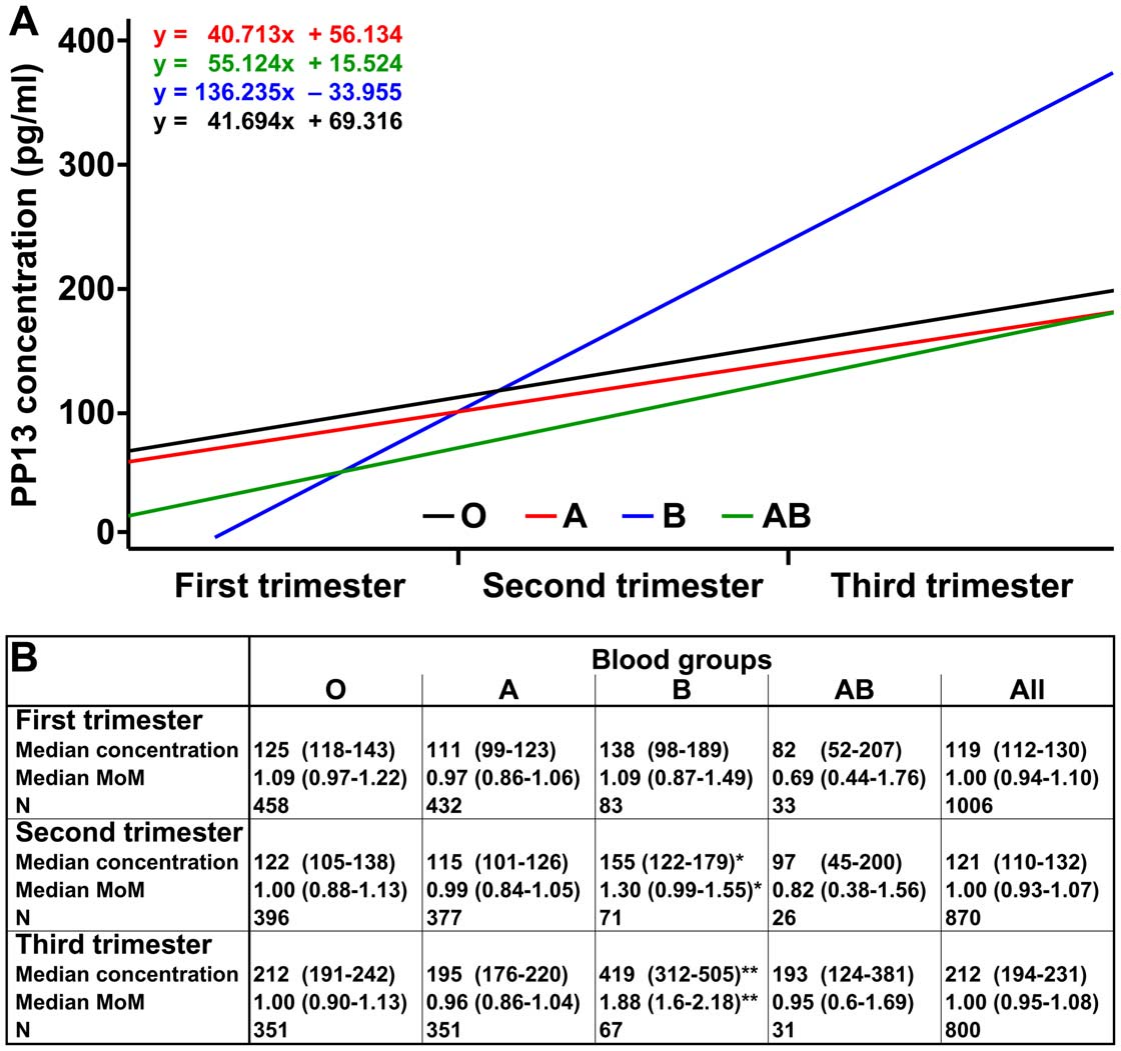
Maternal serum PP13 changes according to maternal ABO blood groups in Caucasian pregnant women. (A) Linear regression analysis was performed for median maternal serum PP13 concentrations (pg/ml) in unaffected women in the study by Gonen et al. [21]. The slope of the regression line (fitted on the medians) was steeper in blood group B than in blood groups A (P = 0.019) and O (P = 0.024). (B) Median PP13 concentrations and median PP13 multiple of the medians (MoMs) (both provided with +/−95% CIs) were compared among unaffected women with various blood groups in the three trimesters. Median PP13 MoMs were calculated after converting gestational-age specific PP13 medians to MoMs and then step-wise adjusting it to BMI, smoking, ethnicity, maternal age and parity but not to ABO blood groups. For statistical analysis, median PP13 concentrations and median PP13 MoMs in each blood group were compared to blood group A by the Wilcoxon rank-sum test; *P<0.05, and **P<0.001. The distribution of PP13 medians and median MoMs were significantly different among the four blood groups in the first, second and third trimesters with a P value of <0.05, <0.05, and <0.001, respectively (Kruskal-Wallis test).

Among unaffected women, maternal serum PP13 concentrations (expressed in pg/ml before adjustment) increased with advancing gestation in all ABO blood groups. The regression slope of PP13 concentrations across the three trimesters was steeper in blood group B than in blood groups A (P = 0.019) and O (P = 0.024), but not in blood group AB (Figure 1A). Similarly, the regression slope of PP13 MoMs (adjusted to 6 confounders) across the three trimesters was steeper in blood group B than in blood groups A (P = 0.020) and O (P = 0.008), but not in blood group AB. Of note, the regression slope in blood group AB ran below the regression slopes in all other blood groups when comparing either PP13 concentrations or MoMs. Regression slopes of PP13 concentrations or MoMs did not differ according to maternal Rh status (data not shown).

When comparing the data in the three trimesters separately, we found that 1) women with blood group AB had the lowest median PP13 MoM in the first trimester, while median PP13 MoM in this blood group was similar to those in blood groups O and A in the third trimester, and 2) women with blood group B had the highest median PP13 MoMs throughout pregnancy (Figure 1B).

#### Changes in maternal serum PP13 concentrations and MoMs according to maternal ABO blood group in Hispanic women [19]

To validate these observations, we re-analyzed the Hispanic cohort data. Among controls, PP13 MoM was also the lowest in blood group AB and the highest in blood group B in the first trimester (Table 1). Similar to the Caucasian cohort, PP13 concentrations or MoMs were not different between Rh+ and Rh− women (data not shown).

**Table 1:** First trimester maternal serum PP13 concentrations and MoMs in Hispanic women.

	Blood groups				
	O	A	B	AB	All
**PP13 conc. (pg/ml)**	89 (56–150)	114 (54–188)	183 (64–310)	60 (13–69)	96 (55–185)
**PP13 MoM**	0.94 (0.50–1.53)	1.01 (0.57–1.81)	1.57 (0.84–3.45)*	0.58 (0.11–0.95)	0.99 (0.51–1.75)
**N**	141	76	20	5	242

Values are presented as medians (interquartile range) or number of patients. Because of the small number of subjects in blood group AB, 95% confidence intervals could not be provided. Median PP13 concentrations and median PP13 MoMs in each blood group were compared to blood group O by the Mann-Whitney test;

* P<0.05.

### PP13 binds to maternal and fetal RBCs *in vivo*

**Figure 2:**
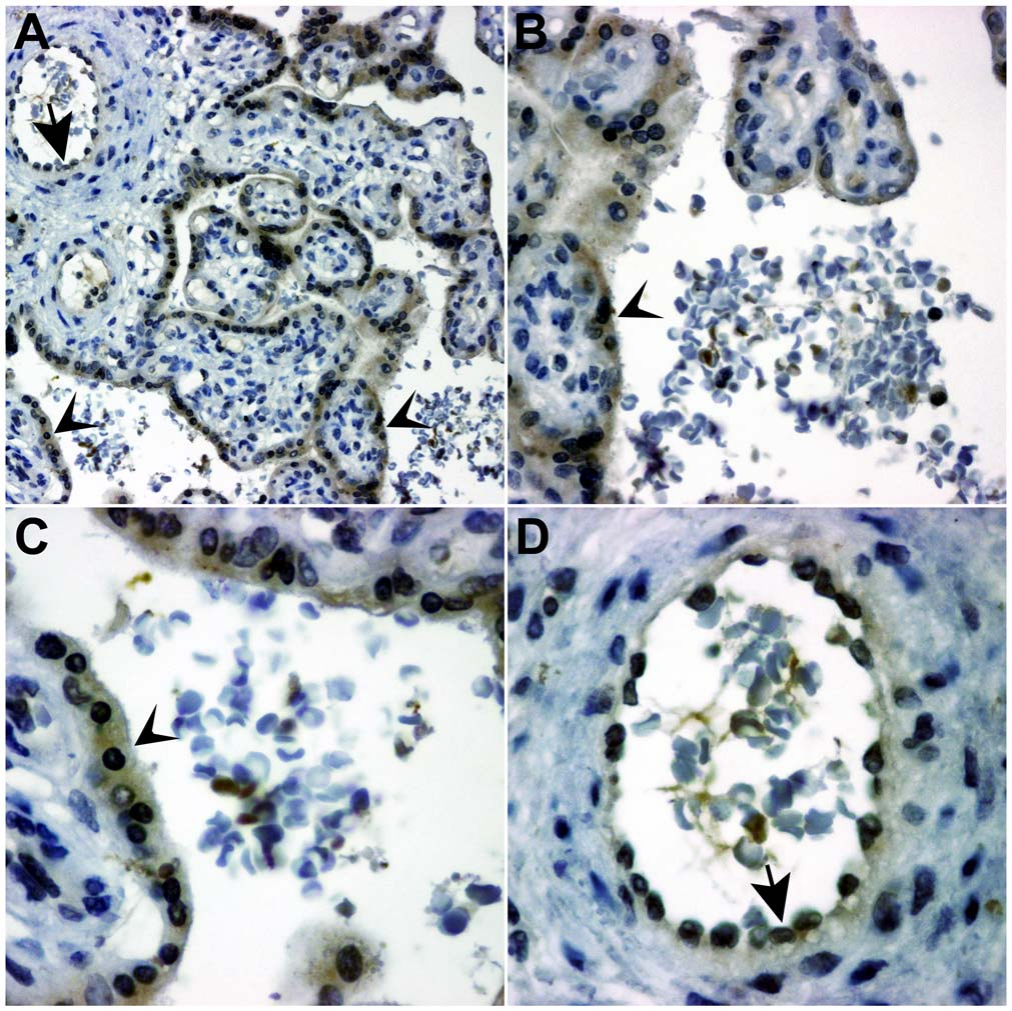
PP13 binds to erythrocytes *in vivo*. Immunohistochemical staining was performed for PP13 in tissue microarrays of normal, term placentas. Besides immunostaining of the syncytiotrophoblast (arrowheads) and the fetal endothelia in the villi (arrows) described earlier [28], [29], [33], [34], PP13 immunostaining was also observed for fetal (A,D) and maternal (A,B,C) RBCs. Different magnifications of the same representative tissue microarray core (A: 200×; B: 400×; C: 630×; D: 630×); hematoxylin counterstain.

To test whether PP13 binds to RBCs *in vivo*, TMAs of normal term placentas were immunostained for PP13. Similar to earlier data [28], [29], [33], [34], the syncytiotrophoblast and endothelial cells of fetal vessels, unique sources of PP13 [29], were stained in all specimens. Although endothelial cells carry ABO antigens, we were unable to evaluate their PP13-binding regarding ABO blood-groups or disease status because of their PP13 expression. Of note, PP13 staining of fetal and maternal RBCs was also found, suggesting that PP13 binds to these cells. Interestingly, not all RBCs were stained for PP13, and the PP13 immunostaining intensity varied between immunopositive RBCs in each specimen (Figure 2).

### PP13 has a differential binding to RBCs of different ABO blood types *in vitro*

**Figure 3:**
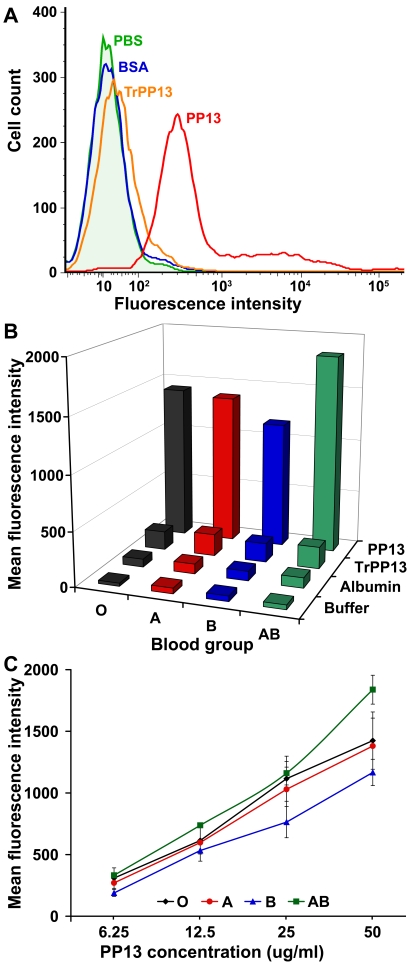
PP13 differentially binds to erythrocytes of distinct ABO blood groups *in vivo*. Erythrocyte-binding assay was run with recombinant PP13, truncated PP13 (TrPP13), bovine serum albumin (BSA) and buffer (PBS), and quantified with flow-cytometry. A) PP13-binding to RBCs was specific and mediated by its CRD, as trPP13 bound negligibly to RBCs, similar to BSA. B) PP13 bound to blood-group AB RBCs with the strongest affinity and to blood-group B RBCs with the weakest affinity (data presented for 50 ug/ml PP13 concentration). C) PP13-binding to RBCs of different ABO blood groups dynamically changed according to the applied PP13 concentrations, similar to that observed for other galectins [37], [38]. Mean values of mean fluorescence intensities (±SEM) are presented from five independent experiments that were run in triplicate.

**Figure 4:**
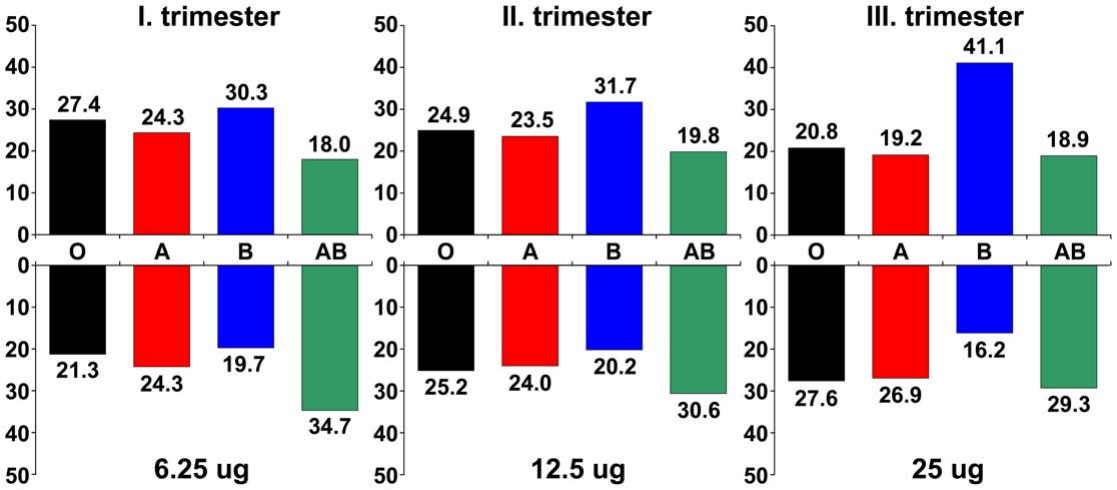
PP13-binding to erythrocytes inversely mirrors serum PP13 concentrations in different ABO blood-groups. The proportional level of median serum PP13 concentrations in unaffected women with various blood groups in the Caucasian cohort is presented in percentiles for the three trimesters, respectively (upper panel). The proportional PP13-binding affinities of RBCs with various blood-types obtained from mean fluorescence intensities are presented in percentiles for three applied PP13 concentrations, respectively (lower panel). The relative PP13-binding to RBCs of different ABO blood types dynamically changed in the chosen protein concentration range and inversely mirrored the relative serum PP13 concentrations in women with different ABO blood-groups with advancing gestation from the first to third trimesters.

To reveal differential binding, we incubated PP13 and control proteins with four ABO blood-type RBCs. PP13-binding to all blood-type RBCs was detected, while BSA and trPP13, a truncated protein that lacks the functional CRD of PP13, had minimal binding to RBCs, proving that PP13-binding was specific and mediated by its CRD (Figure 3A). Consistent with its differential binding to sugars on terminal positions of ABO blood-group antigens [28], [29], PP13 had differential binding to RBCs according to ABO blood types. PP13-binding was similar in blood groups A and O, the weakest in blood group B, and the strongest in blood group AB in comparison to other blood groups (Figure 3B). As with other galectins [37], [38], PP13-binding to various blood-type RBCs dynamically changed with increasing PP13 concentrations (Figure 3C) and inversely mirrored the changes seen in serum PP13 with advancing gestation and concentrations (Figure 4). The quantity of bound PP13 to individual cells varied within a wide range (1000-fold) in each blood group as with binding of other lectins to RBCs [47]. Senescent RBCs, characterized by smaller size and higher granularity [48], bound 1.5–2-fold more PP13 than young RBCs within each blood group (data not shown).

### PP13 binds to blood-group H antigen *in silico*

**Figure 5:**
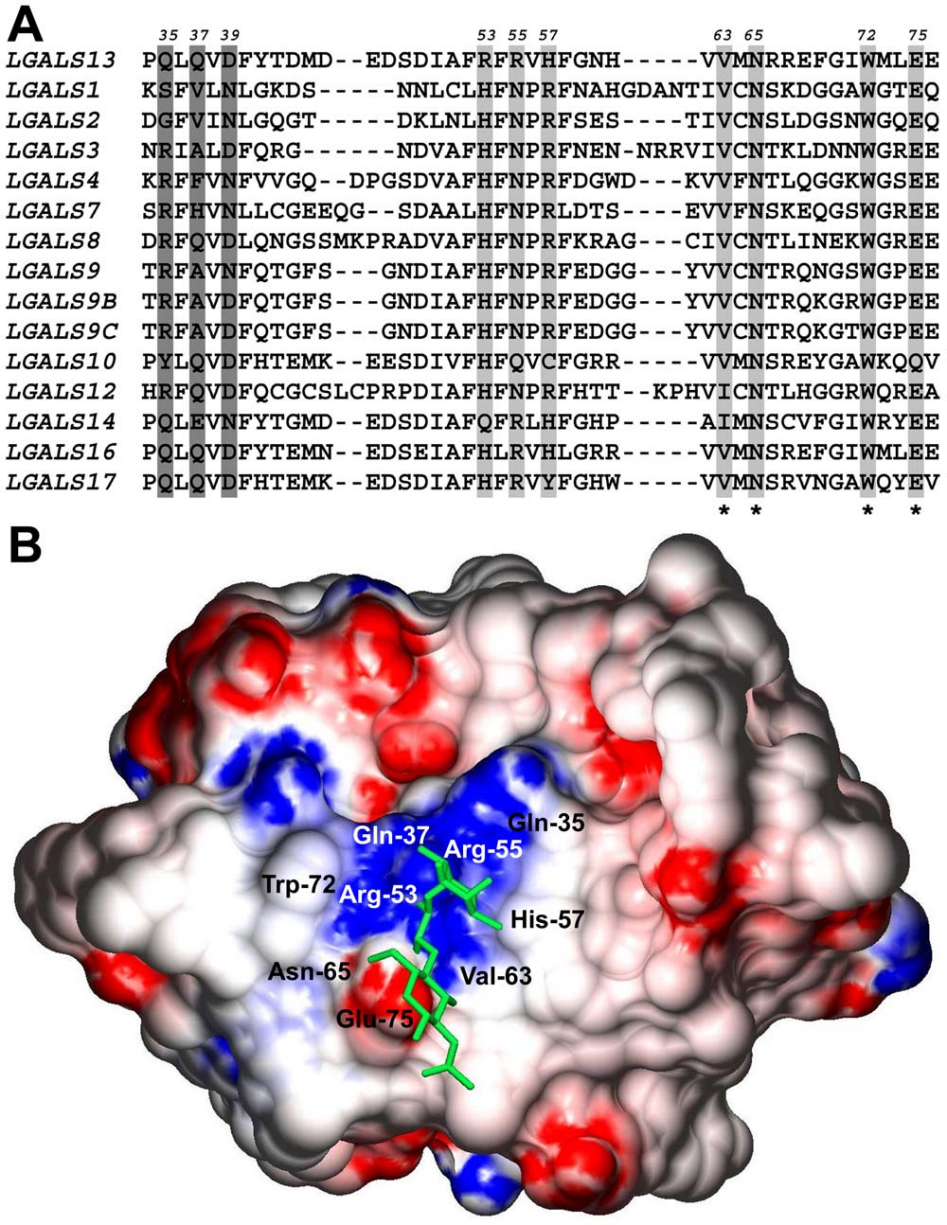
PP13 binds to blood-group H antigen *in silico*. (A) Amino acid sequence alignment of 14 human galectins (partial view). Highly conserved residues in the CRDs that are involved in carbohydrate binding are highlighted in light gray, conserved residues in PP13 CRD are denoted with asterisks. B-sites that are involved in blood-group antigen binding are highlighted with dark gray. Amino acid positions in PP13 are shown above the sequences. (B) Surface representation of PP13 complexed with blood-group H trisaccharide (stick representation). Blue and red indicate positive and negative electrostatic potentials mapped to the molecular surface, respectively. As in CGL2, the binding groove of the PP13 CRD contains a central positive channel flanked by negative regions.

Multiple sequence alignment revealed that out of seven conserved residues in human galectin CRDs, four are conserved in PP13 (Figure 5A). Three of these four residues form the core binding-site [27], [29], while residues in the opposing side of the CRD, which have been under positive selection in PP13 [27], [29], form a positive binding groove. The B-site in PP13 CRD resembles B-sites in human galectins, which participate in blood-group antigen binding [38], [39]. Structural alignment revealed that the structural similarity of PP13 [27] to fungal galectin CGL2 [46] is high (TM-score = 0.77), suggesting that the same oligosaccharides, such as blood-group antigens [46], may be bound by their CRDs. Indeed, our 3D modeling revealed a very similar accommodation of blood-group H trisaccharid in PP13 CRD as in CGL2 CRD [46] (Figure 5B).

### Prediction of pregnancy complications is improved by including ABO blood group in the test

Using the Caucasian dataset [21], we re-evaluated the performance of the PP13 test in predicting pregnancy complications after the adjustment of PP13 MoMs to maternal ABO blood groups. In this cohort, the frequency of ABO blood groups was not significantly different in women with preeclampsia compared to unaffected women (Table S1).

PP13 concentrations and MoMs adjusted to six confounders (GA, BMI, ethnicity, smoking, maternal age, and parity) were significantly lower in all disease groups than in unaffected women in the first trimester, while these were significantly higher in all disease groups than in unaffected women in the second and third trimesters. Women with preeclampsia associated with IUGR had the lowest PP13 MoMs in the first trimester and the highest MoMs in the second and third trimesters (Table 2).

**Table 2:** Maternal serum PP13 concentrations and MoMs in Caucasian women.

Study groups	First trimester	Second trimester	Third trimester
**Unaffected**			
Median PP13 concentration (pg/ml)	119 (112–130)	121 (110–132)	212 (194–231)
Median PP13 MoM	1.0 (0.94–1.10)	1.0 (0.93–1.07)	1.0 (0.95–1.08)
Median PP13 MoM after ABO adjustment	1.0 (0.94–1.07)	1.0 (0.92–1.06)	1.0 (0.93–1.06)
N	1006	870	800
**IUGR**			
Median PP13 concentration (pg/ml)	42 (34–59)	146 (112–220)	258 (185–338)
Median PP13 MoM	0.37 (0.27–0.50)	1.38 (0.95–1.87)	1.22 (0.97–1.62)
Median PP13 MoM after ABO adjustment	0.34 (0.28–0.54)	1.40 (0.95–2.04)	1.24 (0.97–1.69)
N	52	46	42
**Preeclampsia**			
Median PP13 concentration (pg/ml)	32 (22–49)	212 (173–265)	394 (344–730)
Median PP13 MoM	0.27 (0.16–0.42)	1.71 (1.57–2.02)	1.82 (1.53–3.52)
Median PP13 MoM after ABO adjustment	0.23 (0.16–0.44)	1.89 (1.62–2.08)	1.84 (1.53–3.21)
N	20	19	15
**Preeclampsia with IUGR**			
Median PP13 concentration (pg/ml)	25 (12–52)	239 (212–271)	398 (254–563)
Median PP13 MoM	0.25 (0.13–0.41)	1.91 (1.68–2.61)	1.62 (1.17–3.00)
Median PP13 MoM after ABO adjustment	0.21 (0.12–0.44)	1.94 (1.66–2.70)	1.65 (1.22–3.12)
N	5	5	5

Median PP13 concentrations and median PP13 MoMs (before and after adjustment to ABO blood groups) (all presented **+/−** 95% confidence intervals) are provided for the four study groups in the first, second and third trimesters. Although most of the patients gave three blood samples during the study of Gonen et al. [21], some of them gave only two; thus, the number of investigated blood specimens decrease from the first to the third trimester.

**Figure 6:**
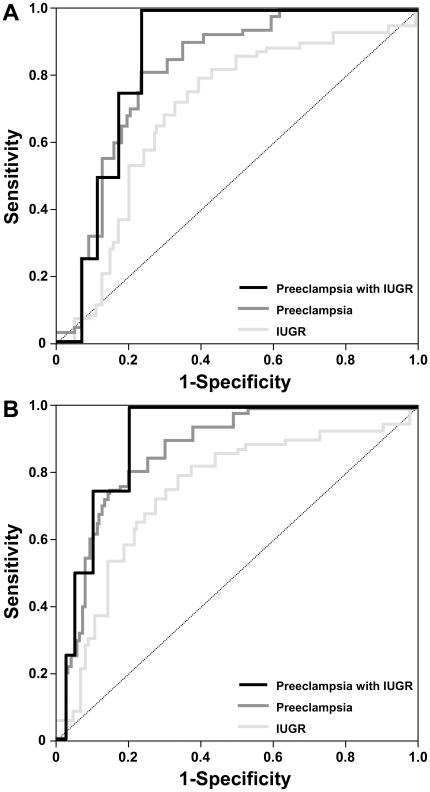
Receiver-operating characteristic (ROC) curves depicting the sensitivity and specificity of PP13 MoM for pregnancy disorders with or without its adjustment to ABO blood groups. ROC curve analysis was used to evaluate the accuracy of PP13 MoM for first trimester prediction of intrauterine growth restriction (IUGR; N = 52), preeclampsia (N = 20) and preeclampsia complicated with IUGR (N = 5) before (A) and after (B) adjustment to ABO blood groups. Areas under the ROC curves (AUCs) for all disease groups were above (P<0.001) the diagonal lines, which represent random prediction. After adjustment to ABO blood groups, AUCs for IUGR, preeclampsia and preeclampsia with IUGR improved from 0.69, 0.81 and 0.85 to 0.75, 0.86 and 0.90, respectively.

First trimester medians of PP13 MoMs in the three disease groups were further lowered after adjusting MoMs to ABO blood groups. In the second and third trimesters, medians of PP13 MoMs in the three disease groups were further raised after adjusting MoMs to ABO blood groups. Blood-group B patients had the highest PP13 MoMs among the disease groups in the second and third trimesters (Table 2). Thus, the adjustment to ABO blood groups increased the differences in PP13 MoMs in all disease groups compared to unaffected controls and improved the prediction accuracy of the PP13 test. In accord, the sensitivities derived from ROC curves (Figure 6) showed an increase of ≤13% for a fixed specificity of 20% false positive rate (FPR) and ≤25% for a fixed specificity of 15% FPR when examined in the first trimester. These differences in sensitivities of the PP13 test after adjustment to ABO blood groups were statistically significant (Table 3). The corresponding increases in areas under the curves (AUCs) after adjustment to ABO blood groups were 6%, 5% and 5% for IUGR, preeclampsia and preeclampsia with IUGR, respectively (Figure 6).

**Table 3:** Sensitivities of first trimester PP13 MoMs before and after adjustment to ABO blood groups.

	Sensitivity(before adjustment to blood groups)	Positive LR	Negative LR	Overall LR	Sensitivity(after adjustment to blood groups)	Adjusted positive LR	Adjusted negative LR	Adjusted overall LR
**15% FPR**								
IUGR (N = 52)	28	1.87	0.85	2.2	53**	3.73**	0.70	5.32**
Preeclampsia (N = 20)	55	3.66	0.53	6.9	75**	6.71**	0.37*	18.1**
Preeclampsia with IUGR (N = 5)	50	3.33	0.59	5.6	75*	7.53**	0.27**	27.9**
**20% FPR**								
IUGR (N = 52)	53	2.65	0.59	4.5	58*	3.99*	0.47	8.5**
Preeclampsia (N = 20)	68	3.4	0.4	8.5	81*	4.91*	0.31	15.8**
Preeclampsia with IUGR (N = 5)	75	3.75	0.31	12.1	75*	5.57**	0.19**	29.31**

FPR: false positive rate, IUGR: intrauterine growth restriction; LR: likelihood ratio. Sensitivities and specificities were calculated from PP13 multiples of medians (MoMs) using receiver-operating characteristic (ROC) curve analysis. The increased sensitivity further lead to increased positive LR (sensitivity/[1-specificity]) and decreased negative LR ([1-sensitivity]/specificity) and an increase in their ratio (overall LR). Note that overall LRs are more than doubled after adjustment to ABO blood group. Significant increases in the sensitivity of the PP13 test after adjustment to ABO blood groups are shown.

*P<0.05,

**P<0.005.

## Discussion

### Principal findings of this study

1) PP13 binds to ABO blood-group antigens on RBCs by its CRD. 2) The differential binding of PP13 to ABO blood-group antigens affects maternal serum PP13 concentrations. 3) Individuals with blood group B have the highest maternal serum PP13 MoM, while those with blood group AB have the lowest PP13 MoM in the first trimester. 4) By adjusting to ABO blood group, the prediction accuracy of the PP13 test is improved for preeclampsia, IUGR and preeclampsia with IUGR.

### ABO blood group confers susceptibility to disease

Glycosylation is the most common post-translational modification in humans, affecting approximately 50–70% of our proteins. Glycans on glycoproteins and other glycoconjugates constitute a complex array termed the “glycome”. Lectins are glycan-binding proteins that decode the high-density “glycocode” stored in the glycome [1], [49], [50]. ABO blood-group antigens are oligosaccharides conjugated to cell-surface glycoproteins and glycolipids or secreted into body fluids by “secretor” individuals [2]. These antigens are synthesized by glycosyltransferases encoded by the H, Se and ABO loci in RBCs, epithelial and endothelial cells, and are also called “histo-blood-group antigens” [2]. The common precursor H antigen is synthesized by fucosyltransferase 1 (H locus) in RBCs and by fucosyltransferase 2 (Se locus) in the secretory epithelium of gastrointestinal and respiratory tracts of “secretor” individuals [2]. The final synthetic step for ABO antigens depends on the ABO locus, which has three major alleles [51]. The A allele encodes alpha-1,3-N-acetylgalactosaminyltransferase, which catalyzes the transfer of N-acetylgalactosamine to the terminal position of the A antigen; the B allele encodes α1,3-galactosyltransferase, placing D-galactose into the terminal position of the B antigen; the O allele harbors a frame-shift deletion, resulting in the synthesis of a protein without enzymatic activity that leaves the common precursor H antigen unmodified [51].

There are six major genotypes and four phenotypes in the ABO blood group with differing frequencies among various populations, which might have been evolutionarily advantageous in conferring resistance against pathogens [3]. Indeed, ABO antigens may alter the presentation of cell-surface glycans and modulate their interactions with pathogens [52] or may provide receptors for pathogen attachment [3]. For example, *P. falciparum* binding to sialoglycans on erythrocytes is indirectly affected by ABO antigens [52]. On the other hand, *C. jejuni* strains directly attach to H antigen, and *E. coli* enterotoxin attaches to A and B antigens in the gastrointestinal tract, while uropathogenic *E. coli* strains bind to A antigen, and *S. saprophyticus* strains bind to A antigen in the urinary tract [3]. In contrast, natural antibodies against ABO antigens can protect the host against pathogens; for example, blood-group B individuals are protected against an *E. coli* (086) that presents blood-group B antigen on its surface [3].

Gastric cancer is also associated with maternal ABO group, having an increased incidence in blood-group A individuals, while blood-group O individuals more frequently have ulcer of the stomach or duodenum [5], [10]. ABO blood-group antigens are linked to the protein backbone of coagulation factor VIII and von Willebrand factor and critically affect coagulation [4], [5]. Indeed, patients with blood-group O are prone to excess bleeding because of the approximately 25% lower plasma concentrations of these coagulation factors [5], which is the consequence of the increased clearance of these glycoproteins, a phenomenon that is related to the H antigen linked to their backbone [5]. Conversely, the elevated plasma concentrations of coagulation factor VIII and von Willebrand factor in non-O blood-group individuals has been implicated in the increased risk for thromboembolic disease and ischemic heart disease [5]–[9]. It was recently suggested that blood group differences in glycosylation of these glycoproteins may alter their interaction with galectins and siglecs, and influence systemic immune functions [53].

### Blood group as a risk factor for preeclampsia

ABO antigens may play a role in the cross-roads of the immune- and coagulation systems by influencing gene-environment interactions. As the “great obstetrical syndromes” [54] (e.g. IUGR, preeclampsia, preterm labor) are characterized by changes in maternal immune- and coagulation systems, differences in ABO blood groups may put a patient at a specific risk according to her inherited antigens. Indeed, large cohort studies identified blood-group AB women at risk to develop preeclampsia [13]–[15]. A population-based case-control study including 100,000 pregnant women revealed that women with blood-group AB were at elevated risk to develop severe preeclampsia (OR: 2.3, 95%CI: 1.3–3.9), early-onset preeclampsia (OR: 3.8, 95%CI: 2.0–7.1), and preeclampsia with IUGR (OR: 3.4, 95%CI: 1.6–7.1) [15]. As the proportion of Caucasian women with preeclampsia and those with blood groups AB and B were low in our study, it was impossible to accurately evaluate the correlation between these blood groups and preeclampsia. The only confirmation that can be derived from our study of the blood-group effect on the risk of preeclampsia is the increase in the significance of the likelihood ratio of developing preeclampsia, particularly preeclampsia with IUGR, following the adjustment of PP13 MoMs to ABO blood groups.

Why would blood group be a risk factor for preeclampsia? An earlier view suggested that inherited thrombophilias may confer increased risk for preeclampsia [55], [56], and increased plasma concentrations of coagulation factors in blood-group AB individuals may have a prothrombotic effect [15], triggering or exacerbating the pathophysiologic events leading to preeclampsia [11]. The current view on preeclampsia suggests that preeclampsia has an exaggerated maternal systemic immune response component [12], [57], [58], and indeed, blood-group antigens influence the bioavailability of E-selectin, TNF-alpha and ICAM1 [59], factors implicated in the pathogenesis of preeclampsia [60]. As galectins are at the cross-roads of the immune and coagulation systems, differences in their bioavailability in different blood groups may suggest a role for galectins in the pathophysiologic regulation of these systems [53], [61].

### ABO blood groups, maternal serum PP13 and preeclampsia

We found ABO blood-group-related differences in maternal serum PP13 in two ethnic populations and *in vivo* and *in vitro* sequestration of this galectin on RBCs, the main sources of ABO antigens in the circulation. Confirming our clinical data, PP13-binding to RBCs inversely mirrored serum PP13 concentrations according to ABO blood groups. PP13 values were almost identical in blood-group O and A women throughout pregnancy as was PP13-binding to blood-group O and A RBCs. Blood-group B women had the highest serum PP13 values throughout pregnancy, and PP13-binding was the weakest to blood-group B RBCs. The lowest first trimester PP13 values were found in blood-group AB women in parallel with the strongest PP13-binding to blood-group AB RBCs.

In this context it is important to note that in the placenta of anthropoid primates PP13 is primarily produced by the syncytiotrophoblast [28]–[32]. This galectin localizes to the cytoplasm and also to the brush border membrane of the syncytiotrophoblast, from where it can be secreted and/or shed into the maternal circulation [28]–[30], [32]–[34]. In normal pregnancies, there is a continuous rise in maternal serum concentrations of PP13 with advancing gestational age [21], [33], similar to the increase in maternal serum concentrations of other proteins synthesized by the syncytiotrophoblast (e.g. Placental Protein 5, alkaline phosphatase, pregnancy-specific beta1-glycoprotein) [62], and similar to the increase in trophoblast cell volumes [63]. Thus, in normal pregnancies, maternal serum concentrations of PP13 primarily depend on the trophoblast volume and the trophoblastic synthesis of PP13 [33].

Of importance, several case-control studies revealed reduced first trimester maternal serum PP13 concentrations in patients who subsequently developed preterm severe preeclampsia [16]–[26]. This can be the consequence of the decreased placental PP13 mRNA expression observed in these patients as early as in the first trimester and throughout pregnancy [33], [35], [36]. This is important since the origins of preeclampsia can be dated back to the very early events in placentation [11], [12], [57], [58], and the reduced first trimester placental expression of PP13, a galectin that may have important immunobiological functions at the maternal-fetal interface [28], [64], may contribute to the early events in the placental pathogenesis of preeclampsia in these patients. In this context, the reduced bioavailability of PP13 in blood group AB women in the first trimester may hypothetically contribute to the early pathophysiologic events at the maternal-fetal interfaces and increase the risk of preeclampsia in these women. This study has also shown that as maternal serum PP13 concentrations increase during pregnancy, these become similar in women with blood group AB to those in women with blood groups A and O in the third trimester. At this phase an exaggerated maternal systemic inflammatory response already dominates preeclampsia [11], [12], [57], [58], and maternal serum concentrations of PP13 and its bioavailability at the maternal-fetal interface may not have a similar effect on the development of preeclampsia compared to the first trimester.

### The structural basis for the differential binding of PP13 to ABO blood group antigens

In the current study we revealed that the differential binding of PP13 to various ABO blood-group RBCs is mediated by the CRD of PP13, consistent with our previous *in vitro* and *in silico* studies [27]–[29] demonstrating the affinity of PP13 to sugars present at terminal positions on ABO blood-group antigens. Importantly, serum PP13 was not affected by Rh antigens, which do not carry glycans. Similarly, several galectins were also demonstrated to bind differentially to various ABO antigens or RBCs carrying various ABO antigens [37]–[39], [46], and ABO antigen-binding was suggested to be mediated by an extended pocket in the CRDs of these galectins [39],[46]. Our sequence alignment and 3D modeling showed that three residues in the core binding-site of galectins which are involved in disaccharide-binding are also conserved in PP13 [27]–[29]. Moreover, the B-site in PP13 CRD resembles to the B-sites of other galectins (e.g. galectin-8), which are involved in blood-group antigen binding [38], [39]. In accord with its overall structural similarity to fungal galectin CGL2 [46], PP13 accommodated blood-group H trisaccharid in its CRD similar to CGL2 [46], suggesting the structural basis for the observed *in vitro* and *in vivo* blood group antigen-binding capability of PP13.

As galectin interactions with oligosaccharides become stronger by cross-linking a large numbers of ligands on cell surfaces [38], [46], [65], [66], the differences observed in PP13-binding affinities *in vitro* and *in vivo* cannot simply be explained by differences in antigen-binding energies between PP13 and its ligands. Other determinants that may also contribute to the differential binding of PP13 to RBCs with various ABO blood types include the following: 1) there is a larger number of A and H antigen-sites compared to B antigen-sites on the RBCs of individuals with the respective blood groups; 2) there is a dynamically changing affinity of galectins to the RBCs with changing lectin concentrations (0.06–10 uM) [37], [39], also found for PP13 (0.175–1.4 uM); 3) the mode of the presentation of glycans on cell-surfaces strongly influences their galectin specificity [37]; and 4) the availability of the B antigen for galectin-binding may be different in blood-group B and AB RBCs due to antigen proximity differences.

Indeed, there is a different localization of ABO blood-group antigen clusters on RBC surfaces since H and A antigen clusters are localized outside or in the periphery of sialylated glycophorin clusters, while B antigen clusters are localized in the center of these sialylated clusters [52]. It is possible that a stronger steric inhibition by sialic acids decreases PP13-binding to B antigens. As indirect evidence for this inhibition, we observed a 1.5–2-fold increase in PP13-binding to “old” compared to “young” RBCs as “old” RBCs lose approximately half of their terminal sialic acid residues [48]. In blood group AB, the close proximity of A and B antigens may be the basis for the stronger binding of PP13 to blood-group AB erythrocytes, leading to its sequestration and lower first trimester serum concentrations, which was also independently observed in cases of preterm preeclampsia, secondary to diminished placental PP13 expression [33], [35]. In light of our findings, we hypothesize that the bioavailability of other galectins that were previously shown to bind ABO blood group antigens [37]–[39], [46] may also be associated with ABO blood groups in the circulation.

### Improvement of the PP13 test for predicting preeclampsia and IUGR

An important outcome of this study is that the adjustment to ABO blood groups further improved the predictive accuracy of first trimester PP13 MoMs for IUGR, preeclampsia and preeclampsia with IUGR. The degree of improvement is not negligible as at false positive rates of 15–20% the adjustment of PP13 MoMs to ABO blood groups improved the detection rate by 13–25%, a change which usually requires the engagement of additional markers into concurrent tests. When further adjusted to ABO blood group, this improvement turned PP13 into a reasonable marker for IUGR, bringing its value to the clinically relevant range for using as a potential predictor. Blood-group adjustment of PP13 MoMs also improved the prediction accuracy for severe preeclampsia (term and preterm combined), complicated by IUGR. This is remarkable since PP13 was earlier shown to be a good marker only for early and preterm preeclampsia [16]–[26]. However, the potential value of the PP13 test for predicting term severe preeclampsia can only be revealed by investigating larger cohorts.

### Conclusions and implications

Our study revealed that ABO blood group affects maternal serum PP13, requiring the addition of blood group as an important confounder in the risk prediction for preeclampsia. This is also the first report suggesting that maternal blood group may be important in the first trimester risk assessment for the subsequent development of IUGR, as well. In light of these findings, we hypothesize that the bioavailability of galectins other than PP13 may also be associated with ABO blood group in the circulation, and we propose that when assaying galectins or other lectins as biomarkers in blood, ABO blood group status need to be taken into account.

Our results showed that there is a greater sequestration and lower maternal serum concentration of PP13 in blood-group AB individuals in the first trimester. Blood group AB, similar to low first trimester maternal serum PP13, is a risk factor for severe preeclampsia. It is possible that the low bioavailability of PP13 in pregnant women with blood group AB in the first trimester contributes to the increased risk of preeclampsia in these patients, and that the coincidence of blood group AB and low PP13 expression may exacerbate the severity of preeclampsia. Although the exact functions of PP13 at the maternal-fetal interface have not been completely discovered, it was recently shown that PP13 can induce apoptosis of activated T cells to a similar extent as galectin-1 [29], a protein implicated in maternal-fetal immune tolerance [67], [68].

## Supporting Information

Table S1 **Patient characteristics in the Caucasian cohort.** *P<0.05, **P<0.01, ***P<0.001 compared to unaffected women in the Caucasian cohort. Values are presented as median (interquartile range)^a^ or number of patients (percentage)^b^.
(DOC)

Table S2 **Patient characteristics in the Hispanic cohort.** Values are presented as median (interquartile range)^a^ or number of patients (percentage)^b^.
(DOC)
