# Supplementary material for: PP13, Maternal ABO Blood Groups and the Risk Assessment of Pregnancy Complications
Source: PLoS One. 2011 Jul 25;6(7):e21564. doi: 10.1371/journal.pone.0021564 (PMC3143125; doi:10.1371/journal.pone.0021564)
Supplement: Table S1 — Patient characteristics in the Caucasian cohort. *P<0.05, **P<0.01, ***P<0.001 compared to unaffected women in the Caucasian cohort. Values are presented as median (interquartile range)a or number of patients (percentage)b. (DOC) [file pone.0021564.s001.doc]

**Table S1. Patient characteristics in the Caucasian cohort**

| **Variable** | **Preeclampsia** | **IUGR** | **Unaffected** |
| --- | --- | --- | --- |
|  | **(N=20)** | **(N=52)** | **(N=1006)** |
| **At enrollment** | | | |
| **Maternal age (years) ^a^** | 27 (25-29)* | 30 (27-33) | 30 (28-33) |
| **<18 years** | 0 (0) | 0 (0) | 1 (0.1) |
| **>40 years** | 0 (0) | 1 (2) | 9 (0.9) |
| **BMI (kg/m^2^) ^a^** | 24.1 (22.7-27.3) | 21.6 (18.6-35.2) | 22.5 (19.2-26.4) |
| **Nulliparity ^b^** | 3 (16) | 19 (36) | 381 (34) |
| **Non-Jewish Caucasian ^b^** | 5 (25)** | 2 (4) | 55 (5) |
| **Previous hypertensive disorders ^b^** | 3 (15)* | 1 (2) | 41 (4) |
| **GA at enrollment (weeks) ^a^** | 7 (6-8) | 7 (6-8) | 7 (6-8) |
| **Smoking ^b^** | 0 (0) | 1 (2) | 59 (6) |
| **Systolic blood pressure (mmHg) ^a^** | 100 (95-116) | 105 (97-113) | 110 (96-116) |
| **Diastolic blood pressure (mmHg) ^a^** | 68.5 (65-72) | 64 (59-72) | 68 (62-73) |
| **At delivery** | | | |
| **Gestational age (weeks) ^a^** | 38 (35-40)** | 38.7 (37.4-40.2) | 39.4 (35-40.3) |
| **Baby birth-weight (grams) ^a^** | 2800 (2500-3400)*** | 2460 (2035-2653)*** | 3320 (2907-3734) |
| **Cesarean delivery ^b^** | 6 (32) | 13 (25) | 195 (17) |
| **Highest systolic blood pressure (mmHg)  ^a^** | 150 (144-161)*** | 110 (99-127) | 110 (98-126) |
| **Highest diastolic blood pressure (mmHg) ^a^** | 90 (85-112)*** | 65 (62-69) | 68 (63- 73) |
| **Proteinuria ^a^** | 2 (2-3)*** | 0 (0-1) | 0 (0-1) |
| **IUGR ^b^** | 5 (25) | 52 (100) | 0 (0) |
| **Blood groups** | | | |
| **Blood group O ^b^** | 9 (45) | 29 (56)* | 458 (46) |
| **Blood group A ^b^** | 8 (40) | 18 (35)* | 432 (43) |
| **Blood group B ^b^** | 2 (10) | 0 (0)* | 83 (8) |
| **Blood group AB ^b^** | 1 (5) | 5 (10)* | 33 (3) |
| **Rh+ ^b^** | 19 (95) | 48 (92.3) | 945 (94) |
