# Supplementary material for: PP13, Maternal ABO Blood Groups and the Risk Assessment of Pregnancy Complications
Source: PLoS One. 2011 Jul 25;6(7):e21564. doi: 10.1371/journal.pone.0021564 (PMC3143125; doi:10.1371/journal.pone.0021564)
Supplement: Table S2 — Patient characteristics in the Hispanic cohort. Values are presented as median (interquartile range)a or number of patients (percentage)b. (DOC) [file pone.0021564.s002.doc]

**Table S2. Patient characteristics in the Hispanic cohort**

| **Variable** | **Normal pregnancy** |
| --- | --- |
|  | **(N=242)** |
| **At enrollment** | |
| **Maternal age (years) ^a^** | 23 (20-28) |
| **BMI (kg/m^2^) ^a^** | 22.3 (20.8-23.7) |
| **<18 years** | 9 (3.7) |
| **>40 years** | 0 (0) |
| **Nulliparity ^b^** | 120 (50) |
| **Hispanic ^b^** | 242 (100) |
| **Previous hypertensive disorders ^b^** | 0 (0) |
| **GA at enrollment (weeks) ^a^** | 10.9 (9.3-12.4) |
| **Smoking ^b^** | 18 (7.4) |
| **Systolic blood pressure (mmHg) ^a^** | 120 (110-120) |
| **Diastolic blood pressure (mmHg) ^a^** | 70 (70-80) |
| **At delivery** | |
| **Gestational age (weeks) ^a^** | 39.9 (39.0-40.4) |
| **Baby birth-weight (grams) ^a^** | 3435 (3240-3630) |
| **Cesarean delivery ^b^** | 19 (8) |
| **Highest systolic blood pressure (mmHg)  ^a^** | 120 (120-128) |
| **Highest diastolic blood pressure (mmHg) ^a^** | 74 (70-80) |
| **Proteinuria ^a^** | 0 (0-1) |
| **IUGR ^b^** | 0 (0) |
| **Blood groups** | |
| **Blood group O ^b^** | 141 (58.3) |
| **Blood group A ^b^** | 76 (31.4) |
| **Blood group B ^b^** | 20 (8.3) |
| **Blood group AB ^b^** | 5 (2.1) |
| **Rh+ ^b^** | 236 (97.5) |
